# A survey of social well-being among employees, retirees, and nursing students: a descriptive-analytical study

**DOI:** 10.1186/s12912-023-01321-w

**Published:** 2023-06-13

**Authors:** Reza Nemati Vakilabad, Roya Kheiri, Negin Islamzadeh, Pouya Farokhnezhad Afshar, Mehdi Ajri-Khameslou

**Affiliations:** 1 RN Students Research Committee, School of Nursing and Midwifery, Ardabil University of Medical Sciences, Ardabil, Iran; 2 School of Behavioral Sciences and Mental Health, Tehran Institute of Psychiatry, Iran University of Medical Sciences, Tehran, RN Iran; 3 Department of Intensive Care Nursing, School of Nursing and Midwifery, Ardabil University of Medical Sciences, Ardabil, RN Iran

**Keywords:** Social well-being, Nurse, Retirees, Nursing students, Social Acceptance

## Abstract

**Background:**

Social well-being is one of the essential dimensions of individual health. Nursing is one of the occupations that can affect a person’s well-being. This study aimed to determine social well-being among employees, retirees, and nursing students.

**Methods:**

This is a cross-sectional descriptive study. 321 samples participated in this study. Convenience sampling method was used to collect samples. Two questionnaires of demographic characteristics and the Keyes Social Well-being Questionnaire were used to collect data. Descriptive statistics, independent t-test, one-way analysis of variance (ANOVA), and Linear regression analysis by the Backward Elimination method were applied using SPSS 14.0.

**Results:**

The mean total social well-being score of this study’s participants was *100 ± 16.43.* The mean social well-being score among nursing employees was *109.58 ± 15.98*, among nursing retirees was *95.67 ± 12.55*, and among nursing students was *93.14 ± 14.81.* Nursing students had lower social well-being scores than nursing employees and retirees *(p < 0.001).* Linear regression analysis showed a significant relationship between the number of children *(p = 0.04) (β = -0.11)*, marital status *(p = 0.04) (β = 2.95)*, and employment status *(p < 0/001) (β = 0.451)* and social well-being and predicted 25% of the total variance of social well-being.

**Conclusion:**

According to the results of this study, the social well-being of retirees and nursing students was significantly lower than nursing employees. Therefore, the educational and healthcare systems of the countries must take the necessary measures to improve the Social well-being of this group of people.

## Introduction

Health is an abstract and multidimensional concept and the simplest definition is the existence of a sense of well-being and the absence of disease [1]. The World Health Organization defines health as complete physical, mental, and social well-being, not just the absence of disease [2]. There are several models of health, but the new perspective is holistic [3]. The holistic model refers to health’s emotional, physical, intellectual, spiritual, psychological, and social aspects [4].

Social well-being is considered one of the aspects of health and a fundamental and essential criterion in a person’s health [5]. Social well-being is the ability to perform social roles effectively and efficiently, evaluating and recognizing how one functions in society and the quality of one’s relationships with others [6]. Social well-being at the individual level includes five components: social integration, social acceptance, social contribution, social actualization, and social coherence [7]. Investigating the social well-being of people in society, especially in the medical field, is of great importance. Low social well-being is associated with depression, reduced social acceptance, isolation, and poor sleep quality [8].

## Background

Various factors such as socioeconomic status, type of job, level of education [9], age, level of social support, and marital status [10] can affect social well-being [11]. Adeb-Saeedi’s (2002) study showed that the hospital environment could harm the social aspect of health [12]. Inequality, labor shortage, and high workload are the most critical problems that can negatively affect the social well-being status of medical staff [13]. Many stressors in the hospital, such as death, severe pain, and emergency situation [14], can adversely affect health workers’ psychological and social well-being [15]. Nurses, constantly exposed to various physical and mental illnesses and difficult working conditions, are at risk of multiple problems in various aspects of health [16]. Various studies show that jobs such as nursing, associated with high job stress, can have devastating physical, psychological, and social effects on nurses [5, 17]. In the study of Shoorvazi, Dalir, Atefi, Tohidi, and Forouhari (2016), the rate of social well-being among nursing employees in Iran has been reported as moderate [18]. Joolaee, Mehrdad, and Bohrani, (2006). Reported that the main reason for the low social well-being of nurses was their negative attitude toward the nursing profession [19]. The type of employment relationship with the hospital and the amount of income affect nurses’ social well-being [20]. This effect is wider than the working period and sometimes continues after retirement.

One of the influential factors that can affect social well-being is retirement [21]. Retirement is one of the most critical and stressful life events in old age that can affect various physical, mental, and social dimensions of elderly health [22]. The study’s results by Andrews et al. showed that nurses experience three significant challenges: work-related stress, lack of flexible working hours, and lack of pay in retirement [23]. Studies have shown that marital status, retirement length, sleep disorder, retirement type (e.g., voluntary or not), subjective cognitive decline, and pre-retirement work conditions (e.g., job strain, occupational complexity, job stress, and burnout) can affect the quality of life of retired nurses [24, 25]. Gabrielle, Jackson, and Mannix (2008) considered the need to assess the health status of nurses in the transition to retirement due to challenging factors in retirement [26]. According to the researchers, the review of the manuscripts indicates that studies have yet to be conducted on the social well-being of nursing retirees in Iran. Also, the studies conducted in other countries on the social well-being of retired nurses are limited. The lack of study on the social well-being of retired nurses indicates the need for more attention from researchers and the healthcare system to the health status of this group of healthcare personnel, which needs further studies.

Another factor that influences social well-being is the type of job. Nursing students have the responsibility to provide health care services in the future. Therefore, their proper social well-being status has a unique role in their efficacy and quality of life. Nursing students are in the hospital during their studies, which can affect their social well-being [6]. Therefore, studying students’ social well-being is particularly important [6, 27]. This issue becomes even more critical when some studies report significant problems in this group, such as entering a larger community, a different educational environment, and new social relationships [28]. Medical students, especially nursing students, face different population segments and various diseases due to high workloads, practical and internship courses in hospitals, and psychological stress caused by the hospital environment [9]. Therefore, having a strong spirit and increased social well-being is a prerequisite for their work to start working in this profession [29]. A study by Salehi et al. (2017) on nursing and midwifery students showed that students’ social well-being was unsatisfactory [9]. In addition, Cicognani et al. (2008) study to evaluate the relationship between social contribution and perception of community among Italian, American, and Iranian students and the effect of these two variables on social well-being showed that the rate of social well-being of American students was higher than Italian and Iranian students [30]. Decreased social well-being among nursing students can have adverse consequences such as academic distress, depressive symptoms, medication errors, and reduced social participation [31, 32]. Studies have shown a significant relationship between well-being and dropout intention [33, 34].

Due to the increase in the number of nursing retirees, the health dimensions of this group are less considered in many communities and studies. At the same time, awareness of this vulnerable group’s social well-being status will help planners make effective interventions. Despite separate studies on the social well-being of each group of nursing staff, nursing retirees, and students, there needs to be more studies on the social well-being of nursing. Therefore, conducting studies such as the present study to examine the social well-being status of three societies related to the nursing profession (employees, retirees, and nursing students) in a study is necessary. This study’s results can show nurses’ social well-being status and the factors affecting it. The results of this study can help improve nurses’ social well-being. Therefore, the present study was conducted to investigate social well-being among employees, retirees, and nursing students and determine the influential factors of the social well-being of nurses.

## Materials

The present study is a descriptive study. The main question of this research was to investigate social well-being among employees, retirees, and nursing students and determine the effective factors of the social well-being of nurses. The study population was employees, retirees, and nursing students in Ardabil. The city of Ardabil is located in northwestern Iran. The total number of nurses in Ardabil was approximately 1000, the number of nursing students in Ardabil was approximately 600, and the number of retirees was approximately 350. According to Cochran’s formula [35], 321 samples were obtained. The convenience sampling method was used for sampling. The research environment was four educational and medical hospitals in Ardabil, the School of Nursing and Midwifery of Ardabil University of Medical Sciences, and the Medical Science Retirees Association. Inclusion criteria include: willingness to participate in the study, ability to speak and read Persian, no mental illness (self-declaration), employment for students, employment in medical wards for nurses, and retirement for retirees; and exclusion criteria include decline continuing research and incomplete filling of the questionnaire.

Data collection occurred between 25 and 2020 and 3 August 2021. Firstly, we informed the head nurses about our study. Written informed consent was obtained from the participants in this study. Questionnaires were distributed among nurses during working hours. Questionnaires were distributed among nursing students of the School of Nursing and Midwifery during office hours, and questionnaires were distributed among retired nurses in the Medical Science Retirees Association during office hours by the researchers (through telephone interviews and face-to-face visits). The completed questionnaires were delivered to the researcher in sealed envelopes. Questionnaires taken from individuals were kept anonymously in a particular file. Then the data were entered into SPSS-14 software by the first author.

Two questionnaires of demographic characteristics and the Keyes Social Well-being Questionnaire (1998) were used to collect data. The demographic characteristics questionnaire included: age, sex, insurance coverage, marital status, number of children, level of education, place of residence, income level, physical activity, addiction, medical history, employment status, length of employment or retirement, and semester.

The Keyes questionnaire has 33 questions that aim to assess social well-being from different dimensions (social actualization, social coherence, social integration, social acceptance, and social contribution) [11]. In this questionnaire, six items are related to social contribution, six items are related to social integration, seven items are related to social acceptance, seven items are related to social coherence, and seven items are related to social actualization. The items are scored on a five-point Likert scale as “Strongly Agree = 5”, “Agree = 4”, “No Opinion = 3”, “Disagree = 2” and “Strongly Disagree = 1”. Therefore, the minimum and maximum scores obtained from this questionnaire will be 33 and 165, respectively. To get the score for each dimension, the total score of the questions related to that dimension was added together and divided by the number of items in that dimension. The total score of all questions is added together to get the total score of the questionnaire. Higher scores will indicate higher social well-being and vice versa. The instrument’s reliability in the study of Cicognani et al. (2008) using Cronbach’s alpha method is 0.88 [30]. In the present study, Cronbach’s alpha method was used to evaluate the reliability of the instrument, with a reliability of 0.85.

Data obtained from Demographic Characteristics Questionnaire and Social Well-being Questionnaire using descriptive statistics (mean, median, standard deviation, etc.) and analytical analysis (independent t-test, one-way ANOVA, Linear regression analysis by Backward Elimination method) were tested using SPSS-14 software. It should be noted that the significant level was considered for statistical analysis as *p < 0.05.* Normality testing of the study variables was also conducted using the Kolmogorov-Smirnov test (*p > 0.05*).

## Results

A total of 321 samples participated in this study, of which 108 were students, 121 were employed nurses, and 92 were retired nurses. The mean age of participants was 35.65 ± 14.24 (Table 1).

**Table 1:** Demographic characteristics of research participants

Variable	Subcategory	N	(%)	Mean (SD) Social Well-being	P-value
Age	19–30	166	51.7	98.33 (18.22)	0.000*
	31–40	57	17.8	109.04 (11.06)	
	41–50	9	2.8	106.33 (21.51)	
	51–60	81	25.2	86.46 (12.8)	
	61–70	8	2.5	89.37 (9.92)	
	Mean 35.65	SD 14.24			
Gender	Female	184	57.3	100.55 (15.95)	0.603*
	Male	137	42.7	99.40 (17.08)	
Marital status	Single	138	43	96.94 (16.50)	0.003*
	Married	183	57	102.42 (16.01)	
Employment status	Student	108	33.6	93.14 (14.81)	0.000*
	Employed	121	37.7	109.58 (15.98)	
	Retired	92	28.7	95.67 (12.55)	
Semester	1–2	23	21.3	85.95 (9.11)	0.000*
(Students)	3–4	27	25	100.88 (14.38)	
	4–6	29	26.9	96.58 (16.49)	
	7–8	29	26.9	88.21 (13.08)	
Work experience	1–8	78	64.5	10.8 (18.60)	0.8*
(Employed Nurses)	9–16	37	30.6	110.32 (8.81)	
	17–24	6	5	106.66 (22.36)	
	Mean 7.64	SD 4.05			
Retirement period	1–4	53	57.6	92.60 (11.01)	0.003*
(Retirees)	5–8	33	35.9	101.45 (13.83)	
	9–12	6	6.5	90.50 (12.55)	
	Mean 4.64	SD 2.18			
Physical activity	Yes	115	35.8	103.58 (17.49)	0.004^±^
	No	206	64.2	98.10 (15.50)	
Residence	City	307	95.6	99.87 (16.34)	0.462^±^
	Village	14	4.4	104.24 (18.27)	
Income	High	70	21.8	99.68 (14.45)	0.945^±^
	Medium	213	66.4	100.28 (16.14)	
	Low	38	11.8	99.55 (16.48)	
Number of children	1	76	45.5	107.77 (15.31)	0.000*
	2	63	37.7	100.31 (15.09)	
	3	24	14.4	95.97 (13.90)	
	4	4	2.4	88.50 (4.65)	
Under insurance coverage	Yes	283	88.2	100.39 (16.50)	0.336^±^
	No	38	16.8	97.65 (15.88)	
Underlying Disease	No	271	84.4	100.42 (16.58)	0.643^*^
	Hypertension and Heart disease	29	9	96.55 (13.20)	
	Diabetes	12	3.7	98.83 (21.8)	
	Orthopedics and disability	9	2.8	102.22 (13.99)	
History of addiction	No	287	89.4	100.85 (16.74)	0.051*
	Smoking	24	7.5	95.62 (11.06)	
	Alcohol	5	1.6	88.80 (14.09)	
	Other	5	1.6	87.40 (11.39)	

*Note.* ^*±*^ Independent-sample t-test *one-way ANOVA

The relationship between social well-being score and employment status of study participants was significant (mean total social well-being score among nursing employees was 109.58 ± 15.98, among nursing retirees 95.67 ± 12.55 and among nursing students 93.14 ± 14.81 (p < 0/001) (Table 1). According to the statistical analysis results, in addition to employment status, the relationship between age, marital status, semester, retirement period, physical activity, and the number of children was significant with the total social well-being score (p˂0.05) (Table 1).

The mean total score of social well-being of nurses participating in the study was 100 ± 16.43 (Table 2). According to the study results, the highest score among the social well-being subscales was related to social participation 3.45 ± 0.59 and the lowest score was related to social acceptance 2.41 ± 0.65. The relationship between social well-being dimensions and participants’ employment status was significant, and students had lower scores in all dimensions of social well-being than employed and retired nurses, except in the social acceptance dimension, where students scored higher (2.26 ± 0.61) than nursing retirees (2.16 ± 0.41) (p < 0/001) (Table 2).

**Table 2:** Total score and subscales of social well-being among employees, retirees and nursing students

Item	Employment status	Mean (SD)	P-value
Social Coherence	Student	2.95 (0.45)	0.000*
	Employed	3.49 (0.49)	
	Retired	3.07 (0.51)	
	Total	3.19 (0.56)	
Social Integration	Student	3.04 (0.51)	0.000*
	Employed	3.34 (0.51)	
	Retired	3.12 (0.51)	
	Total	3.18(0.53)	
Social Contribution	Student	3.19 (0.612)	0.000*
	Employed	3.77 (0.47)	
	Retired	3.32 (0.51)	
	Total	3.45 (0.59)	
Social Actualization	Student	2.73 (0.64)	0.000*
	Employed	3.31(0.72)	
	Retired	2.89 (0.64)	
	Total	2.99(0.71)	
Social Acceptance	Student	2.26 (0.61)	0.000*
	Employed	2.74 (0.71)	
	Retired	2.16 (0.41)	
	Total	2.41(0.65)	
Total score of social well-being		100 (16.43)	

Note. *one-way ANOVA

Linear regression analysis showed that social well-being was affected by the number of children (p = 0.04), marital status (p = 0.04), and employment status (p < 0/001). People with fewer children were married and employed in the employment category and had higher social well-being scores. These variables predicted 25% of the total variance in social well-being (Table 3).

**Table 3:** Social well-being forecasts based on type of employment, marital status and number of children

characteristics	Unstandardized Coefficients B	Standardized Coefficients Beta	t	P-value	R2
Marital status	13.33	2.12	2.95	0.04*	0.25
Number of children	-2.31	-0.11	-1.53	0.04*	
Physical activity	2.68	0.08	1.08	0.279	
Employment status	-13.14	-0.451	-5.17	0.000**	
Underlying Disease	0.382	0.18	0.24	0.801	

Note. *P < 0/05 **P < 0/001

## Discussion

The aim of this study was to investigate social well-being among employees, retirees, and nursing students. This study’s results showed that the participants’ mean total social well-being score was at the undesired level. Therefore, the highest mean total social well-being score was related to nursing staff. In the study of Mozaffari, Dadkhah, Shamshiri, Mohammadi, and Nayeri (2014), the social well-being status of employed nurses was reported as mid-level to high-level [5]. Whereas, in the study of Shoorvazi et al. (2016), the social well-being status of nurses was reported to be moderate to low [18]. These differences with the present study results can be attributed to the difference between work environment, financial status, and Social interactions. The covid-19 crisis has reduced the social interactions of healthcare workers [36, 37], which can affect the social well-being of nurses. In the present study, the mean total social well-being score of the nursing retirees’ group was 95.67 ± 12.55. According to the literature review, no study has been conducted on the social well-being of retired Iranian nurses. In Afshar, Foroughan, Pirooz, and Ajri (2020). study on military retirees, the social well-being of the group under study was moderate [21]. Therefore, in order to increase the social well-being of the elderly, it is recommended that nursing managers support the creation of social networks among nursing retirees. Also, use the experience of nursing retirees in nursing management. The lowest mean social well-being score was related to nursing students among the participants in this study. In addition, the Key-Roberts (2009) study results on students showed high social well-being status [38]. In the study of Javadi, Darvishpour, Khalili, and Barari (2017), which was conducted to investigate the social well-being of medical students, the average total social well-being score was 79.91 ± 11.88 [6]. However, in the present study, the mean total score of social well-being of nursing students was 93.14 ± 14.81. These differences with the current study results can be attributed to the difference between living situations and working conditions. Iran is under economic sanctions. The financial status of nurses is low, especially in vulnerable groups such as retirees and students. Financial status can affect social well-being [39].

According to the results of the present study, the highest average score of total social well-being on the social well-being scales was related to social contribution (3.45 ± 0.59), and the lowest mean social well-being score was related to social acceptance (2.41 ± 0.65). The relationship between the dimensions of social well-being and the employment status of the participants was significant, and students in all dimensions of social well-being had lower scores than nursing employees and retirees, except in the dimension of social acceptance, where students had a higher score (2.26 ± 0.61) than nursing retirees (2.16 ± 0.41). In Shapiro and Keyes’s (2008) study, the mean score of social contribution was higher than other dimensions of social well-being [15], consistent with the present study. In the results of Keyes (2004) and Lima (2006) studies, the lowest mean score of social well-being among the dimensions of social well-being was related to social coherence [40, 41]. In the studies of Abdollah Tabar, Kaldi, and Salehi (2008) and Javadi (2018), the findings showed that students scored higher on average in terms of social integration [6, 42]. The reason for the high average score of social acceptance of students compared to retirees in this study can be due to differences in the study population in terms of environment (student presence in the academic setting) and the young age of students compared to retirees, which caused students to have high social acceptance compared to the retirees. However, the cause of this case needs further study. In the present study, social acceptance of all three occupational groups was downward, which was not consistent with the studies of Mohammadi, Kheftan, Amirpour, Sepidehdam, and Gholami (2018) and Corey (2004) [40, 43]. The reason for this can be related to the increase in social alienation and decrease in social commitment of study participants during covid-19 pandemic, which requires further studies in line with the present study.

The study’s findings showed a significant negative relationship between the retirement period and the average total social well-being score of retirees. Seyfzadeh’s (2015) study on the population of non-nursing elderly showed that there was a statistically significant relationship between the retirement period and social well-being status [44]. However, Saeid, Makarem, Khanjani, and Bakhtyari’s (2019) study on the elderly living in nursing homes showed no significant relationship between the retirement period and health [45], which was inconsistent with the present study. With increasing age and duration of retirement, factors such as social performance, social support, and physical strength decrease, and by reducing these factors, social well-being status is significantly affected [44, 46].

The present study showed a significant relationship between nursing students’ mean total social well-being score and the semester. Thus, the highest average social well-being score was related to the third and fourth semesters, and the lowest average score was related to the first and second semesters and the seventh and eighth semesters. The study of Javadi (2017), which was conducted to assess the social well-being of students, had the highest mean score of total social well-being related to the fourth and seventh semesters and the lowest mean score of social well-being related to the sixth semester [29]. The low average score of the total social well-being of students in the first and second semesters can be due to entering a new academic environment, distance from the city and family, and insufficient university and dormitory facilities. The increase in the social well-being score of nursing students in the third and fourth semesters is related to the growth in social interactions and the formation of student groups. In addition, final-year students’ low average social well-being scores can be due to concerns about future careers and entering the hospital environment as a nurse.

According to the results of the present study, there is a significant relationship between marital status and the participants’ mean total social well-being score. The average score of total social well-being of married people was better than single people. The study of Shapiro (2008) in the United States and Afshar (2020) in Iran showed that there was a significant relationship between marital status and the mean total score of social well-being [21, 47], which were consistent with the present study. In a study conducted in the Netherlands, no statistically significant difference was found between the social well-being status of married and single individuals [48], which was different from the present study. In the current study environment, according to the cultural context of the society, single people have less social activity than married people. It is logically expected that the emotional and psychological needs of married people will be better met than those of single people. Therefore, they may have better social and psychological well-being [21].

Various studies have shown the effect of physical activity on health, but few studies have examined the relationship between this factor and social well-being [49]. In the present study, there was no significant relationship between the mean total social well-being score and physical activity in regression analysis. However, in the analysis of the t-test, this relationship was significant. In their study, Farzi, Zardoshtian, and Eidipour (2015) reported high social well-being among physically active [50]. Physical activity and individual and team sports play a vital role in preventing lifestyle-related diseases, promoting health, and improving psychological and social functioning [51]. Forming sports teams and doing team sports can increase social well-being among nurses, retirees, and students.

The work environment is one of the critical determinants of various dimensions of health, and with increasing work experience, living together and the consequences of the work environment increase [52]. Considering that many people surveyed did not have the necessary mobility in their work environment and personal life, changing their behavior and lifestyle and making sports a part of their lives seems essential. Therefore, nursing managers are recommended to prepare strategies to encourage employees, retirees, and nursing students as an important group among the medical staff to participate in sports activities and create the necessary facilities for them.

Finally, the relationship between social well-being and the participants’ employment status was significant; students had lower scores on social well-being than nursing employees and retirees. Healthcare systems should implement programs such as increasing social participation, and self-awareness, reducing the atmosphere of punishment and blame, strengthening friendships, and creating a culture of appreciation to promote the social well-being of nurses, especially retirees and nursing students.

### Limitations and advantages

Similar to other studies, the present study has some limitations. Firstly, we used a self-administered questionnaire to collect data, which may cause bias. Secondly, our study was limited in budget and time. Accordingly, we conducted the study in only one city. Therefore, the present study’s findings need more generalizability. Future studies should be conducted on the social well-being status of employees, retirees, and nursing students in other areas with consecutive multicenter studies. Another limitation of this study was the convenience sampling method, which suggests that subsequent studies use a random sampling method to select samples. This study is a limited part of the studies conducted on the social well-being of nurses in Iran. The results of this study showed the unfavorable social well-being of retirees and nursing students in Ardabil City.

## Conclusion

Our study’s results showed that the social well-being rate of retirees and nursing students was significantly lower than nursing employees. Also, single nurses and nurses with more children had less social well-being. This means that their social well-being deserves special attention in nursing students and retirees than nursing employees. As a result, practical strategies to promote social well-being, especially ​​social acceptance, must be developed and implemented. These results can be considered by the authorities to provide the basis for the promotion of the dimensions that have obtained the lowest average score, and with the necessary planning and provision of the essential arrangements, can improve the social well-being status of employees, retirees, and nursing students. Nurses’ social well-being requires that basic and higher-level needs are met at the individual/ community and organizational levels. Therefore healthcare systems of the countries must take the necessary measures to improve the well-being and occupational health of this group of nurses. According to the present studies, the effect of physical activity and the promotion of social and collective activities on the social well-being of nurses can be measured in the form of an experimental study.

## Data Availability

The datasets generated and analyzed during the current study are not publicly available due to the participants’ dissatisfaction and the institution. However, they are available from the corresponding author upon reasonable request.
